# Insights from the p53 induced TIGAR protein 2 in the glycolytic pathway model

**DOI:** 10.6026/97320630018310

**Published:** 2022-03-31

**Authors:** Mohammad Jahoor Alam

**Affiliations:** 1 Department of Biology, College of Science, University of Hail, Kingdom of Saudi Arabia

**Keywords:** p53, TIGAR, Glycolytic pathway, numerical simulation, Oscillation

## Abstract

TIGAR is a p53 inducible gene that triggers changes in glycolytic metabolic pathway states. It is known that TIGAR expression lowers the fructose - 2, 6-bisphosphate levels resulting in an inhibition of glycolysis and decrease in intracellular ROS levels. Therefore, it is interesting to document data on p53 induced TIGAR protein 2 in the glycolytic pathway. We describe a two-oscillator model consisting of the p53-Mdm2 network and glycolytic pathway with the TIGAR protein. The numerical simulation of the model shows the suppression of glycolytic oscillation as the level of TIGAR protein increases in agreement with the experimental results reported. Thus, stochastic simulation data helps to understand the realistic behaviour in the pathway.

## Background:

The biological rhythms play essential roles in many cellular processes. They can emerge as the collective dynamic behaviour of an ensemble of interacting components in the cell [1]. Examples include oscillations in glycolysis in which the enzyme phosphofructokinase-1 might be considered as the primary oscillophore and the enzymic basis of glycolytic oscillation [2]. The tumor suppressor p53 protein is considered to be one of the most important hubs in the biological network which influences most of the important biological functions. In normal or unstressed cells, p53 levels are kept low through a continuous degradation of p53. MDM2 (also called HDM2 in humans) plays a major role in the control of p53 [3,4]. Moreover, p53 plays as a transcription factor for MDM2 protein. Other proteins, such as Pin1, are recruited to p53 and induce a conformational change in p53, preventing Mdm2-binding. After p53 stabilization, p53 switches on the expression of target genes. These target genes drive a variety of cellular responses to stress, including DNA repair, cell-cycle arrest, senescence, apoptosis and genome stability [5,6]. p53 protein has a direct role in cellular metabolism. P53 identifies the product of a p53 target gene, TIGAR (TP53-induced glycolysis and apoptosis regulator), and shows that it alters the pathway in which a cell uses glucose [7-10]. Recently, it has been reported that TIGAR has functional similarities with bisphosphatase domain (FBPase-2) of the PFK-2/FBPase-2 (6-phosphofructo-2-kinase/fructose-2,6-bisphosphatase) enzyme[11-15]. Moreover, the PFK-2/FBPase-2 enzymes ubiquitinate fructose-2,6-bisphosphate (Fru-2,6-P2). Further, Fru-2,6-P2 induced 6-phospho-1-kinase to convert fructose-6-phosphate to fructose-1,6-bisphosphateasthethird step of glycolysis [16-18]. Again, Fru-2,6-P2 slows down the formation fructose-6-phosphate. Similarly, TIGAR causes a decline in Fru-2,6-P2 levels and thereby blocks glycolysis [19-22]. Glycolytic dynamic behaviour involves a first-order source step for generating a substrate for PFK-1, which is experimentally simulated by the substrate injection technique [23-28]. Under many conditions, the enzyme pyruvate kinase might follow passively the oscillating pace set by the enzyme PFK-1, allowing perfect synchronization of the whole process [29-32]. Therefore, it is interesting to document data on p53 induced TIGAR protein 2 in the glycolytic pathway.

## Material and Methods:

The two-oscillator model of p53-Mdm2 network and glycolysis pathway is constructed based on various experimental reports and describes our model's detailed reaction mechanisms. We then describe the methods of our simulation of the reaction model briefly. In our model, we assume that p53 is synthesized at a constant rate and that under normal conditions; it is usually bound to Mdm2 and then degraded. It is reported that p53 is only transcriptionally active when not bound to Mdm2, so the production of Mdm2_mRNA depends on the pool of unbound p53. Thus Mdm2_mRNA provides the intermediary link between p53 and Mdm2 to provide the necessary delay in the negative feedback loop. We also include the degradation of Mdm2 and Mdm2_mRNA.P53 induces the production of TIGAR protein. As the TIGAR has functional similarities with bisphosphatase domain (FBPase-2) of the PFK-2/FBPase-2 enzyme, it directly interacts with PFK-2/FBPase-2 enzymes, which leads to decrease the formation of fructose-2,6-bisphosphate (Fru-2,6-P2). Also, Fru-2, 6-P2 slows down the formation of fructose-6-phosphate by controlling PFK-1 enzyme. The molecular species and reaction channels that are involved in this pathway are listed in Table 1 and Table 2(see PDF), respectively.

## Mathematical model of two-oscillator network:

If we denote the molecular species by a vector X=[X1, X2, X3, X4, X5, X6, X7, X8] where, X_1=p53, X_2=Mdm2, X_3=Mdm2_p53, X_4=Mdm2_mRNA, X_5=TIGAR, X_6=Fru-2,6-P2, X_7=Fru-2,6-P2_TIGAR, X_8=Fru-1,6-P2 respectively are the molecular concentrations. Using Mass action law the set of reactions in Table 2.2(see PDF) B can be represented by the following set of ordinary differential equations: (see PDF)

These are classical deterministic non-linear differential equations which can be solved by various numerical techniques. We used Fourth order Runge-Kutta method of numerical integration to solve the set of ordinary differential equations (1)-(8)(see PDF). The plots of our simulated data are carried out in Xmgrace plotting software which is already inbuilt in Linux operating system.

## Results:

We present the numerical simulation results of the model. The plots in Figure 2(see PDF) showed the oscillatory dynamics of p53 which induce TIGAR protein activate and then affect the dynamics of 1-6 bisphosphate. Initially, as the concentration of TIGAR is small due to p53 network (e = 0.0001), it does not affect much the dynamical oscillatory behaviour of 1-6 bisphosphate. However, if the TIGAR concentration is lower (e = 0.001) then the amplitude of the 1-6 bisphosphate is significantly reduced and at some particular value of it (e > 0.05) the oscillation is almost suppressed (Figure 3 - see PDF). This gives rise that the induced TIGAR protein regulates the fate of the glycolysis whose increase in concentration above a threshold the glycolytic process is suppressed which is in agreement to experimental reports. Next, we present the two-dimensional results showing the behaviour of various proteins, p53, Mdm2, 1-6 bisphosphate (Figure 4 - see PDF) for a constant value of e. The plots show the oscillatory behaviour of TIGAR induced by p53 oscillation. The oscillation in 1-6 bisphosphate is due to a negative feedback loop in the glycolysis process. We then show the results of the regulation of 1-6 bisphosphate for different concentration levels of TIGAR in the network (Figure 5 - see PDF). The results show the diminishing of the oscillatory amplitudes of 1-6 bisphosphate as a function of TIGAR concentration levels.

The dynamics of p53, TIGAR and 1,6bisphosphate as a function of time for different values of e are shown in Fig. D. Because of the increase in e the level of TIGAR is also increased with increase in amplitude. Due to the increase in TIGAR concentration level, the oscillatory behaviour exhibited by the negative feedback loop of the glycolysis network is suddenly decreased. Thus, the glycolysis mechanism is controlled by TIGAR concentration level.

## Discussion:

The two oscillatory models were built to study the relation between p53 protein and glycolysis metabolism. P53 is highly studied for its role in various metabolism in both normal and cancer cells. Moreover, the glycolysis process is very high in cancer cells as the cancerous cells need more energy to grow [33-36].Our numerical simulation results of the proposed model indicate that when the intrinsic concentration of the p53 protein is deficient, the concentration level (shown by "e") of TIGAR protein is shallow, as shown in Figure 1(see PDF) as the TIGAR synthesis is directly dependent on the p53 protein [37-40]. Moreover, in the glycolytic oscillatory pathway, the 1,6 bisphosphate also follows a normal oscillatory behavior, as shown in the last panel Figure 2(see PDF). The oscillatory behaviour of molecular species involved in the p53 network is documented in Figure 3(see PDF), which shows the stress in the p53 network. Moreover, we followed the 1,6 bisphosphate concentration within the cell at the various time frames and TIGAR concentrations. It is observed that 1,6 bisphosphate amplitude diminishes as the TIGAR concentration increases with respect to an increment of time frame supported by various experimental reports [22,41]. Finally, numerical simulation result suggested an encouraging effect of the p53 protein oscillatory network upon the glycolytic oscillatory network via TIGAR protein. Further, TIGAR can be a possible target protein for the therapeutic intervention of cancer cells [42, 43].

## Conclusion:

We documented interference ofp53 induced TIGAR protein 2 in the glycolytic pathway. Further, as the TIGAR protein 2 plays an essential role in checking the glycolysis pathway, the study will benefit in molecular therapeutics and drug design studies and strategies for controlling the glycolytic pathways in normal and cancerous cells. Further, the cellular dynamics are stochastic due to the various molecular interactions; therefore, we need to take up stochastic models to see how noise affects the dynamics.

## References

[1] Lanfang L (2021). J American Heart Association.

[2] Zhou W (2019). Front Mol Neurosci..

[3] Kim J (2015). Am J Physiol Renal Physiol..

[4] Yoshifumi O (2019). Am J Physiol Heart CircPhysiol.

[5] Cho Y (1994). Science.

[6] Espinosa JM (2001). Molecular Cell.

[7] Xu H (2019). Sci Rep..

[8] Won KY (2012). Hum Pathol.

[9] Romeo M (2018). Virology.

[10] Norden E (2019). Carcinogenesis..

[11] Mondal P (2021). FASEB J..

[12] MadanE et (2012). Br J Cancer..

[13] Lee P (2015). Cell Death Dis.

[14] Kim SH (2016). Pathol Res Pract..

[15] Kimata M (2010). Am J Physiol Heart Circ Physiol..

[16] Jiang X (2019). J Cell Mol Med.

[17] Iwao C, Shidoji Y (2015). Biomed Res.

[18] Hoshino A (2012). J Mol Cell Cardiol.

[19] Green DR (2006). Cell.

[20] Dai Q (2013). Int J Biochem Cell Biol.

[21] Bensaad K (2006). Cell.

[22] Al-Maghrebi M (2016). Peer J.

[23] Bensaad K (2006). Cell.

[24] Lu X (2005). CurrOpin Genet Dev..

[25] Cawley S (2004). Cell.

[26] Liu Z (2019). Front Oncol.

[27] Wei CL (2006). Cell.

[28] Nemoto S (2004). Science.

[29] Vogelstein B (2000). Nature.

[30] Vousden KH (2006). J Cell Sci.

[31] Liu G (2006). J Cell Biochem.

[32] Bensaad K (2005). Nat Med.

[33] Sablina AA (2005). Nat Med.

[34] Stambolsky P (2006). Cell Death Differ.

[35] Hardie DGJ (2004). Cell Sci.

[36] Jones RG (2005). Mol Cell.

[37] Imamura K (2001). Biochem Biophys Res Com..

[38] Feng Z (2005). Proc. Natl. Acad. Sci. U. S. A..

[39] Malik MZ (2017). Mol Biosyst..

[40] Mathupala SP (1997). J Bioenerg Biomembr.

[41] Smith TA (2000). Br J Biomed Sci.

[42] Tian M (2005). Ann Nucl Med.

[43] Mathupala SP (1997). J Biol Chem.

